# Calibration-free reaction yield quantification by HPLC with a machine-learning model of extinction coefficients†

**DOI:** 10.1039/d4sc01881h

**Published:** 2024-05-29

**Authors:** Matthew A. McDonald, Brent A. Koscher, Richard B. Canty, Klavs F. Jensen

**Affiliations:** a Department of Chemical Engineering, Massachusetts Institute of Technology Cambridge Massachusetts 02139 USA kfjensen@mit.edu

## Abstract

Reaction optimization and characterization depend on reliable measures of reaction yield, often measured by high-performance liquid chromatography (HPLC). Peak areas in HPLC chromatograms are correlated to analyte concentrations by way of calibration standards, typically pure samples of known concentration. Preparing the pure material required for calibration runs can be tedious for low-yielding reactions and technically challenging at small reaction scales. Herein, we present a method to quantify the yield of reactions by HPLC without needing to isolate the product(s) by combining a machine learning model for molar extinction coefficient estimation, and both UV-vis absorption and mass spectra. We demonstrate the method for a variety of reactions important in medicinal and process chemistry, including amide couplings, palladium catalyzed cross-couplings, nucleophilic aromatic substitutions, aminations, and heterocycle syntheses. The reactions were all performed using an automated synthesis and isolation platform. Calibration-free methods such as the presented approach are necessary for such automated platforms to be able to discover, characterize, and optimize reactions automatically.

## Abstract 2

Reaction optimization and characterization depend on reliable measures of reaction yield, often measured by high-performance liquid chromatography (HPLC).

## Graphical abstract

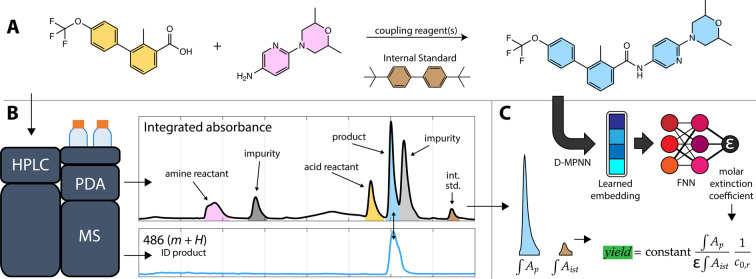

## Introduction

Yield determination is critical for the discovery of new reactions,^1^ optimization of complex transformations,^2,3^ and establishment of reaction scope.^4^ Laboratory automation tools are now routinely used to discover and optimize reactions, and high-throughput systems have increased the number of reactions tested by scaling down to micro/nano-moles of material in 96-, 384-, and even 1536-well plates.^2,5–7^ At this scale, determining the yield of a reaction is difficult without having reference material. Even at gram-scale, yield determination can be tedious with the most common methods not being amenable to automation.^8,9^ An ideal solution would require minimal sample preparation and use common laboratory equipment. High-Performance Liquid Chromatography (HPLC) has become a workhorse for medicinal and process chemistry laboratories^10^ and for analyzing well plates prepared by automated laboratory systems.^11–13^

In this study we describe an HPLC-based approach to reaction yield estimation requiring minimal sample preparation and no reference material. The method is targeted towards reactions to produce drug-like molecules and was developed for a chemistry platform that autonomously explores targeted regions of organic chemical space.^11^ The platform executes a variety of organic reactions at the ten-micromole scale, handling multiple 96-well plates simultaneously to complete multistep syntheses of proposed new molecules. To enable fast, automated analysis of these small-scale reactions, the approach uses only a single small-volume sample of crude reaction mixture and circumvents the need for reaction workup, mass balances, calibration standards, and HPLC method development; each of these steps can be lengthy on their own.^9^ The approach leverages two HPLC detectors—a photo-diode array (PDA) and mass spectrometer (MS)—, spectral peak resolution, and a machine-learning (ML) model of molar extinction coefficient (*ε*). While we developed and validated this technique to enable an autonomous chemistry platform it may be generally useful as a means of simplifying reaction yield estimation.

**Fig. 1:**
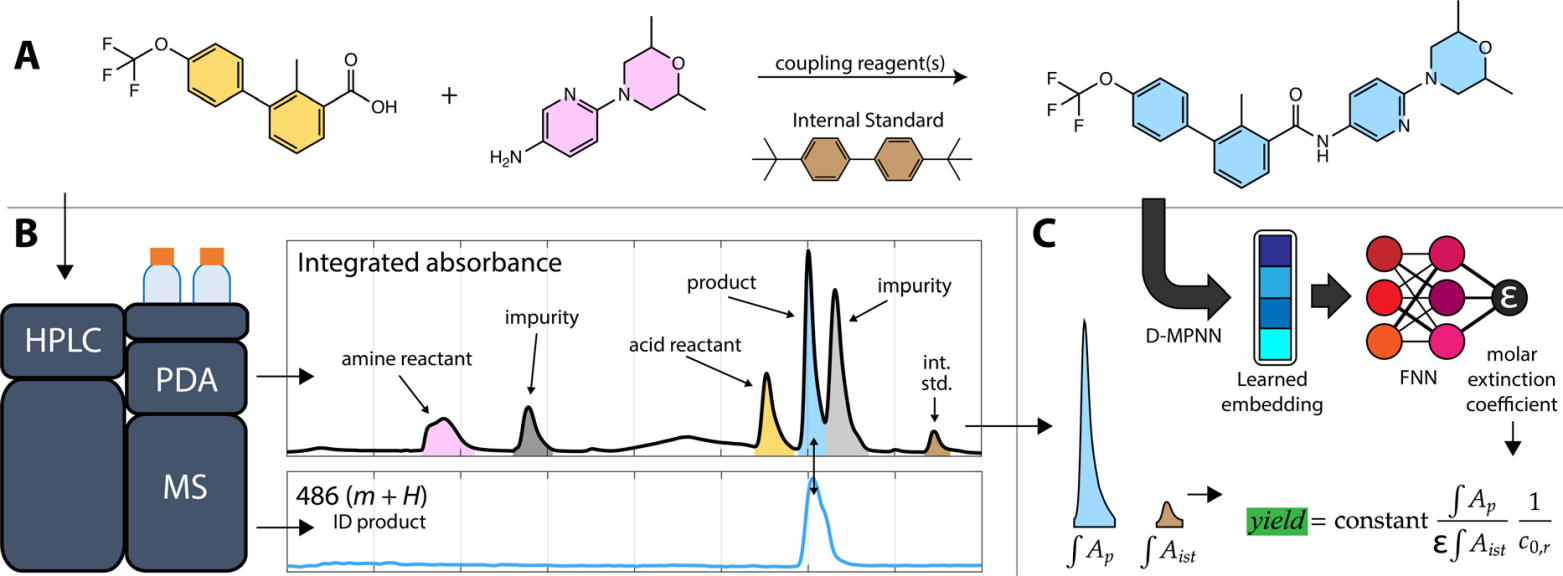
Overview of the calibration-free yield estimation method. (A) The reaction shown is the final step in the synthesis of the Hedgehog signaling pathway inhibitor sonidegib, with molecule shading corresponding to the peaks in the chromatogram in B. (B) A sample of the crude reaction mixture is injected into the HPLC producing MS and PDA chromatograms. The target analyte (sonidegib, blue) peak is identified by the MS chromatogram as the exact mass (485 g mol^−1^) plus a proton (*m* + H). (C) The corresponding peak in the PDA signal is resolved from an overlapping impurity peak (light gray). The area of the resolved peak (∫*A*_P_), the area of the internal standard peak (brown, ∫*A*_ist_), the molar extinction coefficient (*ε*) predicted from a learned embedding, the initial concentration of the reactant (*c*_0*,*r_), and a system constant, derived from the injection volume and detector path length, are used to estimate the reaction yield.

To validate the ML model and proposed method, we instructed the autonomous platform to synthesize a series of drug and drug-like molecules with a variety of reaction plans. The yield of each of the synthesized molecules was estimated with the method and compared to a measured calibration curve based on pure product samples. We also validated the method on a larger series of simulated reactions: solutions of drug and drug-like molecules prepared by the platform in known concentrations. An example reaction (the final step in the synthesis of the Hedgehog signaling pathway inhibitor sonidegib) and the workflow to estimate the reaction's yield is shown in Fig. 1. Overall, we found that the method has similar accuracy to other universal detectors, such as evaporative light scattering (ELSD), with typical yield estimates having less than 20% error on our platform. The error derives from both model uncertainty and automated liquid handling inaccuracy, which can be partially mitigated with internal standards; however, automated reaction discovery, scoping, and optimization at the drug-discovery scale are often tolerant of the method's error rate.

### Method overview

The first step in calibration-free yield estimation is limited sample preparation to ensure compatibility with HPLC. HPLC is an accessible and wide-ranging analytical tool that does not require special facilities nor expertise (compared to NMR or other techniques),^14^ but it does impose some limitations on the types of reactions and reaction products that can be analyzed. As with most HPLC-derived methods, both manual and automatic, our workflow starts by diluting a reaction aliquot in an appropriate solvent, then filtering the sample to ensure that the analyte solution is free of solids that would otherwise clog the instrument plumbing.

A sample of the analyte solution is injected into the HPLC system. In the automated workflow, a custom API is used to interface with the HPLC instrument control software to run samples automatically with an autosampler. The automated process quickly examines the most intense peak in the absorbance data to make sure that the signal is within the dynamic range of the PDA detector and can inject additional samples with varied volumes to ensure a robust signal.

Analysis of the chromatograms involves two data processing steps. First, the product peak is identified based on the known product mass in the MS chromatogram. Then, the corresponding peak in the absorbance data is deconvoluted from baseline and impurity species' signals. Fig. 1 shows each step of the method. The absorbance is proportional to the concentration of the target analyte by the Beer–Lambert law: 
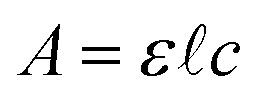
 where *A* is absorbance, *ε* is the molar absorption coefficient, 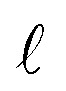 is the path length of the PDA detector, and *c* is the concentration of the analyte. Integrating this equation over the entire peak leads to a linear relationship between the peak area (*a*) and the total number of moles of injected analyte: 
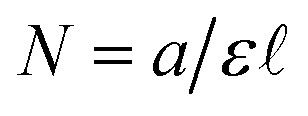
. This linearity is often used to build calibration curves, which avoids the need to measure the molar absorption coefficient and can account for irregular baselines and peak overlap of impurity species. Herein, we demonstrate an ML model that can predict the molar absorption coefficient coupled with resolution of the analyte spectrum from baseline and impurities' spectra to avoid the need to build a calibration curve. Instead, the yield of a reaction, or concentration of an unknown analyte, can be directly estimated with a single HPLC-MS sample.

### Extinction coefficient model

The molar absorption coefficient model was trained using chemprop, a popular Python package for making predictions of molecular properties.^15^ Chemprop employs a directed message passing neural network (D-MPNN) to learn task-specific molecular representations (from molecular graphs input as SMILES strings) followed by a feed-forward neural network (FNN) to estimate properties using the learned representation. Learning accurate features requires sizable training datasets. Fortunately, recent results have shown that co-training on related tasks and pairing the learned representations with calculated features can improve model performance in data-sparse applications.^16^ Since molar absorption coefficients are wavelength dependent (and are typically measured at the reddest absorption peak to avoid overlap with UV-absorbing impurities/solvents), we co-trained models to predict molar extinction coefficient and reddest peak wavelength simultaneously (see the ESI† for training details). Co-training improved the model mean absolute error (MAE) by 12% compared to training on the extinction coefficients alone.

**Fig. 2:**
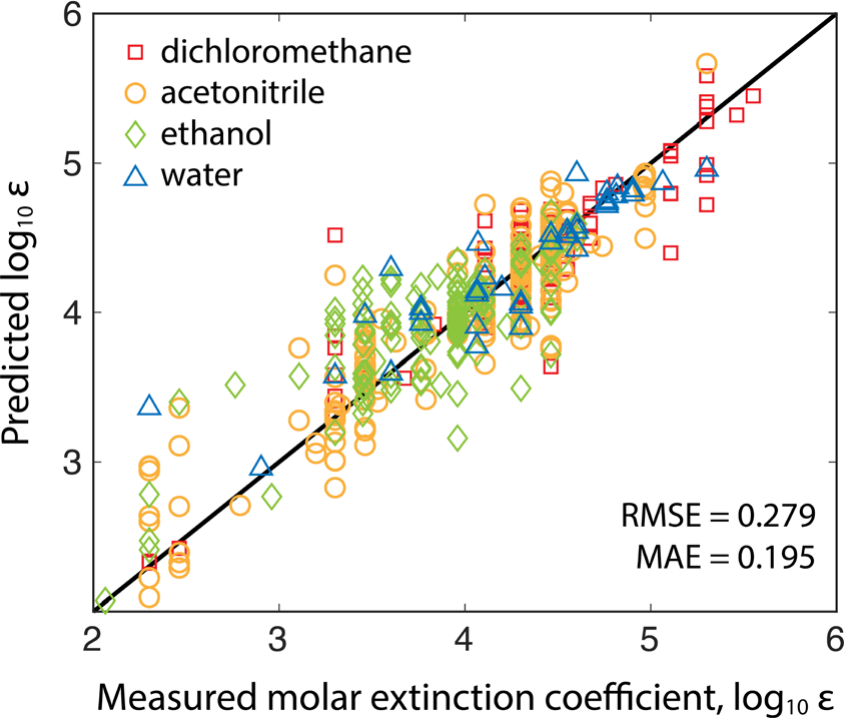
Validation of the model trained on the Reaxys 38k dataset. The predicted *versus* measured log_10_ molar extinction coefficients of 1000 test molecules excluded from both datasets are plotted with marker shape/color indicating the measurement (and prediction) solvent.

Two models were trained on two different training sets: the publicly available Deep4Chem database of 3800 molecules,^17^ and a proprietary dataset from Reaxys of 38 000 unique measurements (Reaxys registry numbers are provided in the ESI†). The model takes as input the target analyte and the solvent because extinction coefficients and absorption peaks depend on the molecular environment. The Deep4Chem database was compiled with a focus on optically active materials (dyes, fluorescent probes, *etc.*) whereas we desired a model of more general organic molecules. Therefore, when creating the 38k dataset, we used data for organic molecules weighting less than 800 g mol^−1^ with extinction coefficient measurements in common HPLC solvents. Table 1 shows a comparison of the two models with 10-fold cross-validation on the same 1000 molecule test set covering the four most common solvents (breakdown of solvents used in training in ESI†). The 38k training set outperforms the Deep4Chem model, with root-mean-square error (RMSE) and MAE both about 40% better, and the variance between folds (standard deviations of the RMSE and MAE) indicating that the model trained on the larger dataset has higher average confidence and better coverage of more diverse chemical structures. While RMSE is always more sensitive to outlying errors than MAE, the differences between RMSE and MAE within each model indicates substantial error stems from a handful of outlying points. When using the model, an ensemble of predictions are made from each of the 10 cross validation models, and the variance in the ensemble of predictions is used as a proxy for model uncertainty.^18^ Moving forward, we use the Reaxys 38k dataset (Fig. 2), but the same method can be implemented with the Deep4Chem dataset, albeit with slightly lower confidence. More model details, including hyperparameter optimization, are included in the ESI.†

**Table 1:** Summary of molar extinction coefficient model performance on 1000-molecule test set over 10-fold cross-validation

10-Fold X-valid.	Deep4Chem 3.8k	Reaxys 38k
Average MAE	0.367	0.195
Std. dev. of MAE	9.70 × 10^−3^	2.86 × 10^−3^
Average RMSE	0.511	0.279
Std. dev. of RMSE	11.8 × 10^−3^	4.81 × 10^−3^

### MS data processing

We developed this method to determine the yield of known transformations applied to new reactants and therefore know the structure of the expected product and its exact mass. The retention time is determined using the MS chromatogram and the mass charge ratio (*m*/*z*) of the expected product based on known ionizable functional groups and adducts. The MS detector uses an electrospray- and atmospheric pressure chemical-dual ionization source (Shimadzu DUIS) and the *m*/*z* channels are automatically adjusted each run for increased sensitivity to the target *m*/*z* ratio. With the molecular structure, the molar absorption coefficient can be estimated using the ML-model; however, the solvent to be used for the prediction depends on the gradient composition when the peak elutes. All our samples are run with a consistent water–acetonitrile gradient (gradient details are in the Sample preparation section). The molar absorption coefficient is estimated by making predictions for pure acetonitrile and water and taking their weighted average based on the volumetric gradient composition at the retention time indicated in the MS chromatogram. The change in polarity with composition of a binary mixture of solvents is generally nonlinear,^19^ but for water and acetonitrile the relationship is nearly linear on a volumetric basis so the weighted average can be applied.^20^ The eluent first passes through the PDA followed by the MS, introducing a delay that was empirically corrected. After aligning the chromatograms, the product absorption peak can be identified with high confidence based on temporal overlap and shape similarity to the *m*/*z* peak; peak intensity was not considered because ionizability and absorption are uncorrelated.

### PDA data processing

The PDA detector generates two-dimensional data consisting of spectra collected between 200–800 nm over the duration of the HPLC run. Before any quantification or peak identification is made, the signal is smoothed, the solvent peak is subtracted, the baseline is corrected, and the chromatogram is scaled according to the internal standard. For baseline subtraction, we found that the asymmetrically reweighted penalized least squares method (arpLS)^21^ was most effective for removing drift associated with solvent composition gradients. Baseline subtraction was necessary for robust peak identification and significantly improved the quantitative accuracy of the method, a review of baseline algorithms for chromatography came to similar conclusions regarding arpLS as a generally applicable baselining algorithm.^22^

For analysis of reactions, in the simplest case, the peaks of each component elute separately, and the yield can be estimated from the area of the product peak. Typically, an HPLC gradient method that results in the product peak being baseline separated from all other species' peaks is desired.^23^ Tailoring methods for individual reactions is an intensive manual task, although it is becoming simpler with continued progress on automated gradient optimization algorithms.^24^ Our method avoids intensive method development by resolving non-baseline separated species based on spectral similarity. The components of the overlapped peaks can be separated provided the species do not substantially co-elute or have indistinguishable absorption spectra. Previous studies have developed a number of methods for resolving spectra, such as parallel factor analysis (PARAFAC), PARAFAC2,^25,26^ and shift-invariant tri-linearity (SIT),^27^ as well as chromatography specific applications such as MOCCA.^14^ These methods were developed for analyzing series of chromatograms, where each chromatogram represents a mixture of the same (sub)set of species. A robust measurement of the absorption spectrum of each species is possible when combining multiple chromatograms, even accounting for shifts in retention time (PARAFAC2) and spectral shape constraints (SIT).

Our method is meant to quickly estimate reaction yield/concentration from a single chromatogram, therefore in this study we resolve peaks by simpler and faster multivariate curve resolution (MCR) using least squares.^28^ MCR involves solving *A* = *CS*^*T*^ + *E* where *A* (*I* × *J*) represents the raw absorption data (over *I* time points and *J* wavelength channels), *C* (*I* × *N*) represents the resolved elution profiles (for *N* components), *S* (*J* × *N*) represents the pure spectrum of each component, and *E* is the error to be minimized. MCR is initially run on the baseline separated peak identified by MS as containing the target species assuming a single component. If the error minimization for the product-containing region is below a predetermined threshold (0.2 AU × *s*) then the peak is assumed to be pure. If the error remains high, additional components are added until the threshold is met, or more than three components are required. We chose three as the maximum number of resolvable components as it was a good balance between accuracy and the duration of our chosen HPLC method. Shorter solvent gradients cause more peaks to overlap and increase the burden of spectral resolution whereas longer gradients can separate more peaks, lessening the need for robust spectral resolution. In principle, the platform could automatically perform an analysis with a longer gradient on samples with more than three overlapping components, but we flagged those samples and withheld them from the analysis (only one of our test reaction systems required more than three components, enalapril overreaction, and is detailed in the ESI†). The target is assigned to the resolved peak with the closest matching MS retention time, which can become a source of substantial error if peaks overlap severely and the wrong assignment is made.

The integral of the intensity of the resolved target over the duration of the chromatographic peak at the wavelength of the reddest absorption peak, is combined with the predicted molar absorption coefficient and the pathlength of the PDA detector 
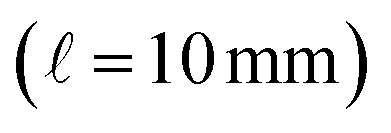
 to estimate the number of moles of the product in the injected sample. This estimate, with the known injection volume, sample dilution volume, and reactant concentration gives the estimated reaction yield. The algebraic details of this calculation are illustrated in the ESI.†

### Sample preparation

The simulated reactions and actual reactions were all prepared by an automated platform developed to discover new molecules with targeted properties by applying known chemical transformations.^11^ While the method could be equally useful to a chemist checking their reactions manually, we chose to test the method with our platform to demonstrate how it can be used to debottleneck reaction analysis in an automated workflow.^29^ The platform uses a Tecan Freedom Evo liquid handler to prepare all samples and a Shimadzu Nexera Series autosampler to inject HPLC samples. A selective compliance articulated robot arm (SCARA) shuttles samples held in 96-well plates between instruments. The liquid handler is equipped with 8 independent pipette channels with Teflon-coated, water-backed, 350 μL tips. The liquid handler can access a library of chemical stock solutions at specified concentrations; these stock solutions were prepared manually in 1.5 mL vials and typically range in concentration from 0.2–2.0 M.

For simulated reactions, between one and three non-reactive chemicals from the library were randomly mixed in dimethyl sulfoxide (DMSO) at a concentration of 5 mM and a total volume of 100 μL. The pipetting action has a typical error of about 0.5 μL (see ESI†), which is insignificant for pipetting nearly 100 μL of solvent but can become significant when dispensing more concentrated stock solutions. To overcome this issue, the stock DMSO was spiked with 4,4′-di-*tert*-butylbiphenyl used as an internal standard. The internal standard peak area was used to normalize all PDA data peak areas and correct for the error associated with liquid handling, the variability in the autosampler injection volume, and the sorption of atmospheric water by dry DMSO while the sample plate sat in the autosampler. None of the targets in the simulated reactions were included in the training set for the extinction coefficient model.

The reactions to form drug and drug-like products were also prepared with the automated platform in 96-well plates. The required reactants were dosed from the platform library and prepared in 200 μL of the designated solvent at 15 mM. For multistep syntheses, working in the retrosynthetic direction, the concentration of each reaction was doubled so that in a two-step synthesis the first step was at 30 mM and the second step was at 15 mM, with the appropriate amount of crude product being dosed into the second reaction based on the estimated yield of the first reaction. After preparation, the well plates were automatically placed on Inheco thermoshakes that heated or cooled the plate to the desired reaction temperature while agitating the reactions with orbital shaking. Palladium catalyzed reactions were prepared and run in a nitrogen-purged box and high temperature reactions were run in an array of 96-well glass vials in an aluminum block. After four hours the reactions were allowed to return to room temperature before being vacuum filtered through a 0.5 μm 96-well filter plate. 50 μL of crude reaction mixture were diluted with 100 μL of internal standard-spiked DMSO and filtered to remove any remaining solids, resulting in samples ready for analysis at approximately the same concentration as the simulated reactions. In this study, all products were sufficiently soluble in DMSO, however failure to dissolve the product will cause the yield to be underestimated.

The samples, including simulated reactions, were automatically transferred to the autosampler which injected 2.0 μL of sample onto a 50 × 4.6 mm, 1.8 μm, reverse phase C18 column. Each run consisted of an eight-minute water–acetonitrile gradient, starting with 0.5 min of 5% acetonitrile, then linearly increasing to 100% acetonitrile over 6 min, holding at 100% for 0.5 min, linearly decreasing to 5% acetonitrile over 0.5 min, then holding at 5% acetonitrile for the final 0.5 min. After analysis of all the samples, any chromatograms with saturated or too little absorption signal were automatically rerun with either half or twice the injection volume, respectively.

## Results and discussion

**Fig. 3:**
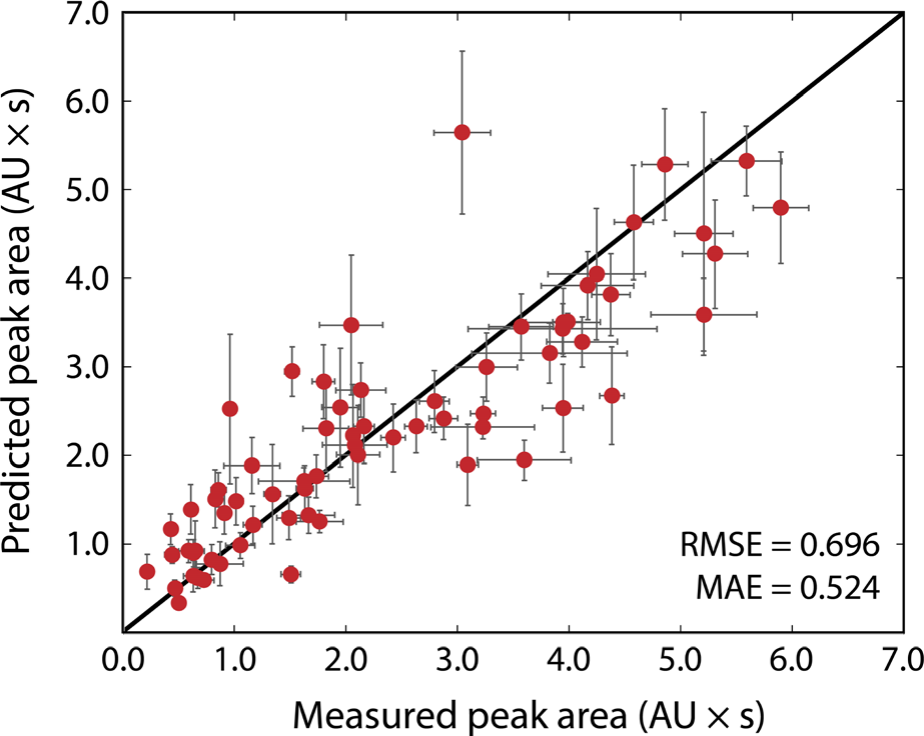
Predicted *versus* measured peak areas, in absorption unit-seconds, for simulated reactions. Error bars on the predicted areas represent the standard deviation of the molar extinction coefficient predictions made with an ensemble of 10 models, propagated into the peak area. Error bars on the measured peak area represent the error from liquid handler pipetting and uncertainty derived from the MCR resolution, some error bars are completely obscured by the data points. The RMSE and MAE evaluated to 0.696 and 0.524 absorption unit-seconds, respectively.

Fig. 3 shows the results of the simulated reactions. The predicted and measured peak areas generally agree well. To predict the peak area, the molar extinction coefficient is predicted with the ensemble of models, and the average value is used along with the known amount injected. The error bars in the predicted values represent the variance in the predicted molar extinction coefficient values. The error bars in the measured peak areas include the uncertainty from MCR peak resolution and liquid handling. Liquid handling was responsible for most of the error, especially for targets that required the liquid handler to dispense fewer than 2 μL to the simulated reaction solution. All simulated reactions have a target concentration of 5 mM, resulting in a dataset that only spans approximately two orders of magnitude since the log_10_ of the molar extinction coefficient only ranges from 2.7 to 4.4; for small organic molecules encountered in drug discovery campaigns this limited range covers most relevant structures. The data in Fig. 3, including the molecules as SMILES strings, are included in the ESI† in tabular form.

The typical error associated with this method is in the 10–25% range (median 18% among simulated reactions). The observed experimental error was found to be moderately correlated with the variance of the ensemble prediction for molar extinction coefficient (Pearson's correlation coefficient of 0.402). This further supports the assumption that model ensemble variance can serve as an indicator for model uncertainty in predicted extinction coefficient values. The prediction ensemble standard deviation can, therefore, inform whether the error of an experiment can be expected to be at the high or low end of the range. The error observed by this method is similar to that from so-called universal detectors like ELSD and Charged Aerosol Detectors (CAD). The accuracy of the presented method can be improved by adding data to the extinction coefficient training set, whereas decreasing the error from a universal detector requires hardware improvements or method redevelopment.

We observed that increasing the number of datapoints by an order of magnitude (3.8k for Deep4Chem to 38k for Reaxys) improved the model performance by about a factor of two (MAE decreased from 0.367 to 0.195). If used in, for example, an experimental campaign wherein the target molecules cover a narrower chemical space, one could train a new model specific to those experiments for improved yield estimation. Studies have shown that a small number of relevant measurements can anchor model predictions to increase accuracy significantly for similar molecules.^30^ The model could also be iteratively updated during the campaign to improve accuracy for later experiments.^11^ The model we present is meant to cover the broad space of small-molecule chemistry and can serve as a useful starting point for targeted campaigns.

The most significant outlier in Fig. 3 is 4-(dimethylamino)-benzoic acid. The chemprop interpretation feature indicates that the dimethylaniline substructure is the most significant factor that led to the nearly 2-fold error in predicted molar extinction coefficient. Dimethylaniline is a very common substructure in diverse families of dye molecules with large molar extinction coefficients (*e.g.*, tetramethylrhodamine as a xanthine dye, methyl orange as an azo-dye, and crystal violet as a triarylmethane dye). The dataset(s) used to train the model is likely biased towards strongly absorbing dye molecules as they are more likely to have their optical properties recorded. Molecules that strongly resemble dyes but do not have dye-like properties, such as 4-(dimethylamino)benzoic acid, are therefore more likely to be overpredicted. At the same time, tests with molecules like indocyanine green, which are engineered to be strongly absorbing, show that the method considerably overestimates the concentration of such strongly absorbing species. Likewise with albumin, a biomolecule far larger than any molecule in the training set, the model underpredicts the molar extinction coefficient (see ESI†). Our model is meant to evaluate reactions for small-molecule drug-like chemistry and cannot be expected to extrapolate well to highly engineered or unfamiliar chemical spaces, such as those represented by indocyanine green and albumin. With data for these types of molecules, a new model could be trained and applied to strongly absorbing dyes or macromolecules.

**Fig. 4:**
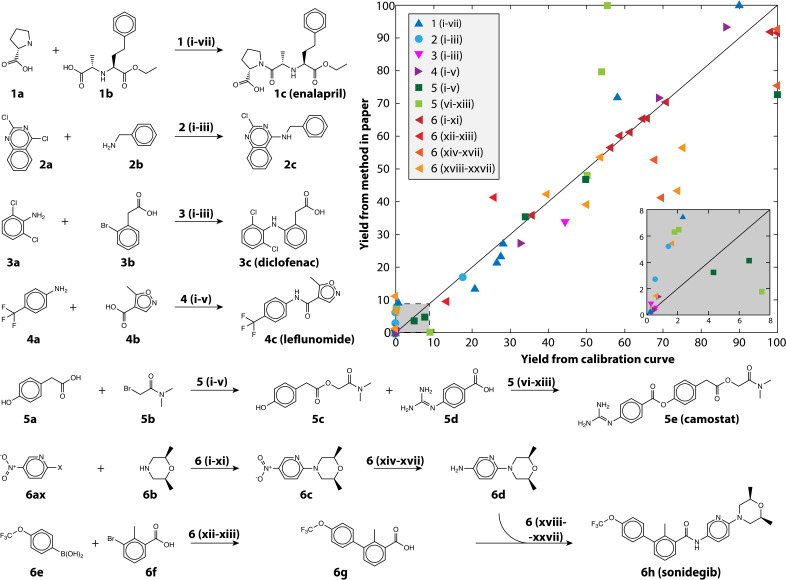
The reactions examined in this study to demonstrate the usefulness of calibration-free HPLC yield estimation. The yield as estimated using the method is plotted against the yield measured with a traditional calibration curve, the inset shows yield between 0 and 8%. The conditions (Roman numerals) for the reaction pathways (Arabic numerals) are given in Table 2.

We also demonstrate the method on a selection of real reactions; simulated reactions cannot account for the complexity of real automated chemistry. We chose reactions in route to six drug and drug-like molecules for which we had pure reference materials to verify yield estimates. The reference materials were used to build calibration curves using the framework developed in MOCCA, an open source chromatographic analysis program.^14^ The reactions represent the variety of transformations that are commonly encountered in medicinal chemistry.^31,32^ The reactions are shown in Fig. 4 and the conditions for each reaction in Table 2; 80 reactions were automatically run and analyzed between the different transformations and conditions tested. The plot in Fig. 4 shows how the method performs at automatically estimating reaction yields based on only a single HPLC run. The markers are colored by the chemical transformation executed (with several different conditions) and the marker shapes indicate which drug molecule was being synthesized. Multistep syntheses, such as the linear synthesis of camostat and convergent synthesis of sonidegib, were conducted with extraction and filtration as the only workup steps between reactions; the final steps in these reactions therefore have numerous peaks from impurities and by-products accumulated in early steps. The yield of these reactions is reported step-wise, and unsurprisingly the performance degrades with increased number of reaction steps.

**Table 2:** Summary of test reaction conditions

Reaction	Temperature	Reagent 1	Reagent 2	Note^d^
1i-ii	40	EDAC	HOBt	Two reactions at 1.0 and 1.5 eq.
1iii-iv	40	HATU, 1.5 eq.	DIPEA, 3.0 eq.	Two reactions, add acid v. amine first
1v-vi	40	CDI, 1.0 eq.	DIPEA, 3.0 eq.^a^	Two reactions, add acid v. amine first
1vii	40	SOCl_2_, 1.5 eq.	DIPEA, 3.0 eq.	
2i-iii	80	KO^*t*^Bu, 3.0 eq.	Pd catalysts^b^	
3i-iii	80	CuI, 0.2 eq.	DBU, DBU, Cs_2_CO_3_, 3.0 eq.	TBAB, aliquat 336, no PTC
4i	40	EDAC	HOBt	Two reactions at 1.0 and 1.5 eq.
4ii-iii	40	HATU, 1.5 eq.	DIPEA, 3.0 eq.	Two reactions, add acid v. amine first
4iv-v	40	CDI, 1.0 eq.	DIPEA, 3.0 eq.^a^	Two reactions, add acid v. amine first
5i-v	80	Bases^c^, 3.0 eq.		Five different bases and eq.
5vi-viii	40	EDAC	HOBt	Two reactions at 1.0 eq., one at 1.5 eq.
5viv	40	PPh_3_, 1.0 eq.	DIAD, 1.2 eq.^a^	
5x-xi	40	CDI, 1.0 eq.	DIPEA, 3.0 eq.^a^	Two reactions, add acid v. amine first
5xii-xiii	40	SOCl_2_	DIPEA, 3.0 eq.	Two reactions at 1.0 and 1.5 eq. SOCl_2_
6i-xi	80	DIPEA	F-, Cl-, and Br-nitropyridine	DIPEA at 1, 2, 3, and 4 eq. each X-pyr
6xii-xiii	80	DBU, 3 eq.	Pd catalysts^b^	
6xiv-xvii	40	SnCl_2_	HCl	At 1.0, 2.0, 5.0, and 10.0 eq.
6xviii-xxi	40	EDAC	HOBt	Two reactions at 1.0, third at 1.5 eq.
6xxii-xxiv	40	HATU, 1.5 eq.	DIPEA, 3.0 eq.	Two reactions, add acid v. amine first
6xxv-xxvi	40	CDI, 1.0 eq.	DIPEA, 3.0 eq.^a^	Two reactions, add acid v. amine first
6xxvii	40	SOCl_2_	DIPEA, 3.0 eq.	Two reactions at 1.0 and 1.5 eq. SOCl_2_

a Reagent added last.

b Palladium catalysts, in order: XPhos Pd G4, tBuBrettPhos Pd G3, (dppf)PdCl_2_.

c Bases, in order, DIPEA, Cs_2_CO_3_, KO^*t*^Bu, DBU, DBU (1.5 eq.).

d Abbreviations. EDAC: 1-ethyl-3-(3-dimethylaminopropyl)carbodiimide, HOBt: *N*-hydroxybenzotriazole, HATU: 2-(7-aza-1*H*-benzotriazole-1-yl)-1,1,3,3-tetramethyluronium hexafluorophosphate, DIPEA: diisopropyl ethyl amine, DIAD: diisopropylazodicarboxylate, DBU: 1,8-diazabicyclo[5.4.0]undec-7-ene, KO^*t*^Bu: potassium *tert*-butoxide, TBAB: tetrabutylammonium bromide, CDI: carbonyl diimidazole, SOCl_2_: thionyl chloride, CuI: copper(i) iodide, PPh_3_: triphenylphosphine, PTC: phase transfer catalyst, X-pyr: halogenated nitropyridine.

The performance is robust across Suzuki couplings, Buchwald–Hartwig aminations, un-activated aminations, amide couplings, aryl and alkyl esterification, nucleophilic substitutions, and reductions, all of which are critical to pharmaceutical discovery and manufacturing, with an overall MAE of 8.3% and RMSE of 12.8% for yield prediction. The reactions themselves span a range of yields, skewed towards lower yield (Fig. 4 inset) because the automated chemistry platform was designed for flexibility and molecular discovery rather than reaction optimization.^11^ The Buchwald–Hartwig aminations failed for several conditions likely because of poisoning by trace water or oxygen. Likewise, the Suzuki couplings did not produce the near quantitative yield that is reported in the literature because the automated platform does not have access to completely air-free conditions, but rather uses a strong nitrogen purge to try and eliminate oxygen. At the same time, simpler reactions such as the aromatic nucleophilic substitution reaction with the fluoro-substituted pyridine (6af) gave nearly quantitative yield in all tested conditions.

Manual inspection of some of the other reactions revealed numerous interesting episodes. The nitro-reduction of 6c with insufficient reductant could produce small quantities of an azo-coupled dimer with very large extinction coefficient. The camostat precursor (5c) could be produced at significantly higher yield using a single equivalent of cesium carbonate and 1.2 equivalents of 5b in dimethylformamide (DMF), whereas reported syntheses use organic bases.^33^ Camostat (5e) itself posed a challenge for the method, as the basicity of the guanidine group causes it to interact strongly with acidic silanol groups present in the column. The extinction coefficient of camostat shifted by 19% and the wavelength of maximum absorptions shifted 15 nm because of the changing solvent composition over the long duration of its elution (see Fig. S3†). Those shifts resulted in MCR identifying additional components at high camostat yields, and caused the strong disagreement seen for the light green squares in Fig. 4.

While in general performance is agnostic of the transformation, there are potential instances when the method can fail because of particulars of the reactants and/or products. When two compounds have nearly identical absorption spectra and strongly overlapped peaks, MCR fails to differentiate them as separate compounds. This was the case for some enalapril syntheses, where more than one proline (1a) was coupled to the starting material (1b), making the yield appear higher than it was (blue triangles in Fig. 4). For the case of enalapril, these compounds could be differentiated by the MS chromatogram (see Fig. S4†), and while the performance on enalapril is still commendable, future improvements to our method could make quantitative use of the MS chromatogram in such instances where absorption spectra are insufficient.

Reliance on mass spectrometry brings its own challenges. In particular, the method cannot differentiate between isomers or isobars, and therefore may not accurately gauge the yield of reactions that are not regio- or stereo-selective. Reaction 2 was chosen to demonstrate the case of regioselectivity as there are two potential amination sites. In this case, we observed small peaks corresponding to both single amination isomers and were only able to differentiate them based on the elution profile of a reference sample. The yield estimate for reaction 2 is very low for both isomers, but had the reaction been higher yielding or strongly site selective the automatically calculated estimate could have been much less accurate. Additionally, as our setup relies on DUIS ionization, the yield cannot be estimated for products that do not ionize under those conditions; for this study we limited the reactions (both real and simulated) to those that included reliably ionizing groups common to drug molecules (such as an amine, acid, heterocycle, or halogen groups). Lastly, the method requires that the product be soluble in an HPLC-compatible solvent and has only been evaluated using reverse phase HPLC running a water–acetonitrile gradient, meaning appropriate solvents must be water-miscible with a polarity between water and acetonitrile. DMF and DMSO are the preferred solvents as they have broad solvating abilities and are non-volatile; however, they absorb atmospheric water that can precipitate more hydrophobic analytes. Normal phase HPLC could eliminate these issues without adversely impacting the calibration-free quantification method.

The error in the yield was also weakly correlated to the error in the predicted absorption maximum (Pearson's correlation coefficient of 0.250), as measured by the PDA detector. This could provide an alternative measure of confidence in the molar extinction coefficient prediction and therefore yield estimate based only on the single HPLC sample. The error in absorption maximum is an immediately available measure of how well the molecule fits the scope of the training data. However, using the error in absorption maximum to give a quantitative estimate of the error in the predicted extinction coefficient will require substantially more work and is beyond the scope of this study.^18^

## Conclusion

We present a method for estimating the yield of a chemical reaction (or concentration of a known analyte in a complex mixture) with a single calibration-free HPLC run. The combination of an MS detector to identify the peak corresponding to the target, an ML model to predict the molar extinction coefficient of the target, and a PDA detector to measure and resolve the absorption peak of the target enable this simple method. We recommend this method for high-throughput discovery, scoping, and screening campaigns where the error typically in the 10–25% range is comparable to universal detectors such as ELSD and CAD; other methods may better suit kinetic data or more thorough reaction investigations.^9,14^ The error could also be improved with more data or campaign-specific data. We demonstrated the method on approximately 80 simulated reactions and an additional 60 small-molecule pharmaceutical relevant reactions. The reactions, simulated and real, were all prepared, executed, and analyzed automatically using a high-throughput platform. Future campaigns to discover new molecules and reactions could be accelerated by enabling automation and reducing tedious reaction analysis.

## Data availability

Details of model training, reaction yield calculation, liquid handling error analysis, simulated reactions, model scope, and reaction outcomes are provided as a PDF in the ESI.† There are numerous additional files and data, hosted on Zenodo at https://doi.org/10.5281/zenodo.10845991. They include: the list of Reaxys registry numbers and assisting python script to retrieve the Reaxys data used to train the 38k model, a python script to simulate running the analysis outside of the automated platform, raw HPLC-MS data for use with the simulation script, data used to make Fig. 2–4, the Deep4Chem dataset (filtered to include only data relevant to this study), trained chemprop model, and test and validation predictions, and the automated platform instructions for running all experiments.

## Author contributions

MAM performed reactions, data collection and analysis, ML model construction, visualization, drafting, and editing. BAK and RBC contributed to reaction automation, data analysis, drafting, and editing. KFJ contributed to data analysis, supervision, drafting, and editing.

## Conflicts of interest

There are no conflicts to declare.

## Supplementary Material

SC-015-D4SC01881H-s001

## References

[cit1] McNally A., Prier C. K., MacMillan D. W. C. (2011). Science.

[cit2] Shields B. J., Stevens J., Li J., Parasram M., Damani F., Alvarado J. I. M., Janey J. M., Adams R. P., Doyle A. G. (2021). Nature.

[cit3] Dunlap J. H., Ethier J. G., Putnam-Neeb A. A., Iyer S., Luo S.-X. L., Feng H., Torres J. A. G., Doyle A. G., Swager T. M., Vaia R. A. (2023). Chem. Sci..

[cit4] Kariofillis S. K., Jiang S., Żurański A. M., Gandhi S. S., Martinez Alvarado J. I., Doyle A. G. (2022). J. Am. Chem. Soc..

[cit5] Cook A., Clément R., Newman S. G. (2021). Nat. Protoc..

[cit6] Taylor C. J., Pomberger A., Felton K. C., Grainger R., Barecka M., Chamberlain T. W., Bourne R. A., Johnson C. N., Lapkin A. A. (2023). Chem. Rev..

[cit7] Gao L., Shaabani S., Romero A. R., Xu R., Ahmadianmoghaddam M., Dömling A. (2023). Green Chem..

[cit8] Berry M. , Silva F., Alimuddin M., Tran-Dube M., Scales S., Yang S., Wang W., Wang F., Sach N. W., Bernier L., Linton M., Ewanicki J. and McAlpine I., presented at the 2023 ACS Fall Meeting, San Francisco, 2023

[cit9] Deem M. C., Hein J. E. (2023). J. Org. Chem..

[cit10] Welch C. J., Gong X., Schafer W., Pratt E. C., Brkovic T., Pirzada Z., Cuff J. F., Kosjek B. (2010). Tetrahedron: Asymmetry.

[cit11] Koscher B. A., Canty R. B., McDonald M. A., Greenman K. P., McGill C. J., Bilodeau C. L., Jin W., Wu H., Vermeire F. H., Jin B., Hart T., Kulesza T., Li S.-C., Jaakkola T. S., Barzilay R., Gómez-Bombarelli R., Green W. H., Jensen K. F. (2023). Science.

[cit12] Farrant E. (2020). ACS Med. Chem. Lett..

[cit13] Grainger R., Whibley S. (2021). Org. Process Res. Dev..

[cit14] Haas C. P., Lübbesmeyer M., Jin E. H., McDonald M. A., Koscher B. A., Guimond N., Di Rocco L., Kayser H., Leweke S., Niedenführ S., Nicholls R., Greeves E., Barber D. M., Hillenbrand J., Volpin G., Jensen K. F. (2023). ACS Cent. Sci..

[cit15] Heid E., Greenman K. P., Chung Y., Li S.-C., Graff D. E., Vermeire F. H., Wu H., Green W. H., McGill C. J. (2024). J. Chem. Inf. Model..

[cit16] Lansford J. L., Barnes B. C., Rice B. M., Jensen K. F. (2022). J. Chem. Inf. Model..

[cit17] Joung J. F., Han M., Jeong M., Park S. (2020). Sci. Data.

[cit18] Heid E., McGill C. J., Vermeire F. H., Green W. H. (2023). J. Chem. Inf. Model..

[cit19] Bosch E., Rosés M. (1992). J. Chem. Soc., Faraday Trans..

[cit20] Gagliardi L. G., Castells C. B., Rafols C., Rosés M., Bosch E. (2007). J. Chem. Eng. Data.

[cit21] Baek S.-J., Park A., Ahn Y.-J., Choo J. (2015). Analyst.

[cit22] Niezen L. E., Schoenmakers P. J., Pirok B. W. J. (2022). Anal. Chim. Acta.

[cit23] Christensen M., Yunker L. P., Shiri P., Zepel T., Prieto P. L., Grunert S., Bork F., Hein J. E. (2021). Chem. Sci..

[cit24] Naser Aldine F., Singh A., Wang H., Makey D., Barrientos R., Wong M., Aggarwal P., Regalado E. L., Haidar Ahmad I. (2024). J. Chromatogr. A.

[cit25] Bro R., Andersson C. A., Kiers H. A. L. (1999). J. Chemom..

[cit26] Kiers H. A. L., ten Berge J. M. F., Bro R. (1999). J. Chemom..

[cit27] Schneide P.-A., Bro R., Gallagher N. B. (2023). J. Chemom..

[cit28] Parastar H., Tauler R. (2014). Anal. Chem..

[cit29] Shi Y., Prieto P. L., Zepel T., Grunert S., Hein J. E. (2021). Acc. Chem. Res..

[cit30] Gómez-Bombarelli R., Wei J. N., Duvenaud D., Hernández-Lobato J. M., Sánchez-Lengeling B., Sheberla D., Aguilera-Iparraguirre J., Hirzel T. D., Adams R. P., Aspuru-Guzik A. (2018). ACS Cent. Sci..

[cit31] Boström J., Brown D. G., Young R. J., Keserü G. M. (2018). Nat. Rev. Drug Discovery.

[cit32] Brown D. G., Boström J. (2016). J. Med. Chem..

[cit33] Ahmed R., Kumar G., Mahajan S., Verma P. K., Singh Cham P., Reddy D. S., Shankar R., Singh P. P. (2023). ChemistrySelect.

