# Supplementary material for: Calibration-free reaction yield quantification by HPLC with a machine-learning model of extinction coefficients
Source: Chem Sci. 2024 May 29;15(26):10092–100. doi: 10.1039/d4sc01881h (PMC11220585; doi:10.1039/d4sc01881h)
Supplement: SC-015-D4SC01881H-s001 [file SC-015-D4SC01881H-s001.pdf]

Electronic Supplementary Material (ESI) for Chemical Science.
This journal is © The Royal Society of Chemistry 2024

Calibration-free reaction yield quantification by HPLC with a machine-learning model of extinction coefficients

*Matthew A. McDonald, Brent A. Koscher, Richard B. Canty, Klavs F. Jensen**

Department of Chemical Engineering, Massachusetts Institute of Technology, Cambridge, Massachusetts 02139, United States

*Email: kfjensen@mit.edu

Contents

Collecting training data and building Reaxys 38k model 2

Model training details and hyperparameter optimization 3

Yield calculation 4

Liquid handling error analysis 5

Simulated reactions details 5

Model scope and outliers 7

Analysis of test reactions 8

Reproducing figures 3 and 4 with provided data and HPLC_analysis.py 10

References 11

Collecting training data and building Reaxys 38k model

The data used to construct the more general model is all available via Reaxys, a proprietary chemical database. To access these data and build a model with the same performance as the one discussed in the main text, requires a Reaxys license. The comma separated value (csv) file “reaxys_registry_numbers.csv” contains all of the Reaxys registry numbers required to query these data and the python file “extract_logE_from_xml.py” can be used to extract the chemical structures and appropriate data fields used to train the chemprop model. To query the data, navigate to the Reaxys “Query builder” tab, select the “Identification” drop down menu on the right, and add “Reaxys Registry Number” (near the bottom of the list) to the query. For the query, copy-paste the Reaxys registry numbers from the csv into the “<> Reaxys Registry Number =” search bar. The query should consist of a list of numbers separated by semicolons (the final entry should not be followed by a semicolon, this will cause query errors). We found that Reaxys occasionally failed on queries of more than 1,000 Reaxys registry numbers, and therefore have broken the input file into 999 molecule chunks, so that you can copy-paste a single column of the csv at a time. It may take several minutes for Reaxys to search the registry numbers, especially if searching more than 1,000 at a time.

After the results are found, export the data as an XML file. To do this, click the “Export” button above the list of results, from the “Choose a format” dropdown select “XML”, for the “Range” select “All results”, for “Export” select “Choose specific data” then “+ Add datapoints”. This will launch a new panel from which you should select the “Spectra” dropdown, then check “UV/VIS Spectroscopy”, and then click “Add datapoints >”. Now back at the export substances window, make sure that “Include structures” is checked and click “Export”. Download the exported data into a common folder, repeating this process for all of the registry numbers in the csv.

The python script "extract_logE_from_xml.py" should now be used to extract the relevant data from the XML files into a new csv file that can be directly used to train chemprop. In addition to some common dependencies, the python script requires RDKit be installed to do some initial screening, formatting, and cleaning of the data (chemprop also requires RDKit, and this script should run cleanly in a chemprop environment). From the terminal, run:

```
python extract_logE_from_xml.py <folder_of_xml_files> <output.csv>
```

The generated output.csv is ready to be used to train the chemprop model. The next section details training the chemprop model, with the "test_set.csv" file corresponding to "output.csv", but with 10% of the data removed to form a validation set. From these data, the breakdown of solvents is 50.7% acetonitrile, 30.7% dichloromethane, 14.5% ethanol, and 4.1% water.

Model training details and hyperparameter optimization

All models were trained using Chemprop version 1.5. The models were trained using the MIT SuperCloud, a Linux environment with Intel Xeon Gold 6248 CPU (40 cores) and a Nvidia Tesla V100 GPU. For reproducibility, the training command line argument was:

```
Chemprop_train --data_path train_set.csv --save_dir model_x\
--num_folds 10 --dataset_type regression --config_path\ hyperopts.json --
epochs 200 --number_of_molecules 2
```

The train_set.csv file consists of four columns of data, the solute and solvent (each encoded as a SMILES), the $\log_{10}$ of the molar extinction coefficient, and the wavelength of maximum absorption (in nm). The configuration file was generated using the built in chemprop_hyperopt command on a subset of 1,000 randomly selected molecules from the training set. Using a subset of the training data significantly decreased the time required for hyperparameter optimization. We

observed that the best validation error occurred at approximately epoch 150, so 200 epochs were chosen to ensure learning completed. The optimized parameters are in the following table.

Table S1. Summary of hyperparameter optimization results

Hyperparameter	Value	Parameter description
depth	3	Number of message passing steps
dropout	0.15	Dropout probability
ffn_num_layers	3	Number of layers in feed forward neural network after message passing
hidden_size	2300	Hidden dimension in feed forward neural network

Yield calculation

The yield is directly proportional to the integral of the absorbance over the specified peak and maximum wavelength ($\int A^{max}$). The proportionality constant depends on instrument parameters, such as the path length of the detector (ℓ), flow rate of the eluent ($\dot{f}$), and injection volume (v_{in}), internal standard true peak area (c_{ist}) and measured peak area ($\int A_{ist}^{257}$), which for 4,4'-di-tert-butylbiphenyl is taken at 257 nm, the initial concentration of the reactant (c_0), and the molar extinction coefficient (ε).

$$yield = \frac{c_{ist}\dot{f} \int A^{max}}{c_0 \varepsilon \ell v_{in} \int A_{ist}^{257}} \times 100\%$$

For the method developed in this study, the internal standard (4,4'-di-*tert*-butylbiphenyl) had a standard concentration of 1.25 mM, resulting in a standard peak area of 0.21 A.U. s/µL (absorbance unit seconds per microliter injected). The flow rate was set to 0.5 mL/min, the injection volume varied depending on the concentration of the reaction, and the initial concentration varied depending on what step in each multistep synthesis was being performed. In general, for each step in the retrosynthesis the concentration of reactants is doubled, starting from 15 µmol per reaction, to account for incomplete yield in early reaction steps. Error bars are

calculated by propagating the uncertainty in the predicted extinction coefficient, calculated as the variance in predictions among the ensemble of models.

Liquid handling error analysis

The uncertainty associated with pipetting via the Tecan Evo LiHa 350 µL pipette tips was measured following the procedure in Bessemans *et al.*[1] A specified amount of DMSO was pipetted into a single well in a 96-well plate, and then the actual amount of DMSO dispensed was measured gravimetrically. This was repeated several times at different volumes to develop a relationship between specified volume and relative pipetting error, shown in figure S1. A power-law was found to best fit the error data, showing that the error increases exponentially at smaller specified volumes, however the power-law is not a perfect fit for the performance of the Tecan Evo LiHa.

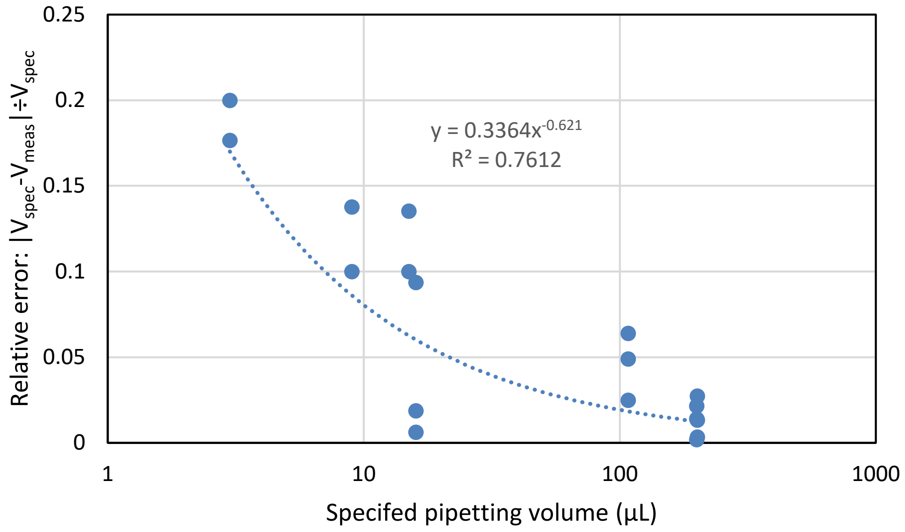

Figure S1. Relative pipetting error versus the specified pipetting volume, plotted on semi-logarithmic axes, over a range of 2 to 200 µL. The dashed curve is a power-law regressed curve.

Simulated reactions details

The data shown in Figure 3, as well as the molecules those data correspond to, are provided in tabular form below.

Table 2. Details of the simulated reactions shown in Figure 3. The molecules are represented as SMILES, with the measured peak area (Figure 3 x-axis), predicted area (Figure 3 y-axis), and accompanying uncertainties.

SMILES	Measured Peak Area (A.U. x sec.)	Predicted Peak Area (A.U. x sec.)	Measurement uncertainty	Prediction uncertainty
O=C(O)c1cccc(O)c1C(=O)O	2.040	3.504	0.285	0.795
COc1ccc(C(=O)O)c(C)c1	2.127	2.818	0.225	0.318
COc1ccc(C(=O)O)cc1OC	4.163	3.928	0.416	0.382
Cc1ccc(C)c(C(=O)O)c1	0.420	1.221	0.042	0.178
O=c1cccc[nH]1	1.792	2.850	0.103	0.415
COC(=O)c1sccc1N	2.870	2.464	0.128	0.244
N#Cc1ccc(Br)cc1N	3.981	3.527	0.132	0.100
CCOC(=O)c1ccc(Br)cc1	1.943	2.590	0.166	0.678
O=CCCc1ccccc1	0.718	0.603	0.090	0.056
COc1cc(Br)c(C(=O)O)cc1O	1.510	3.013	0.067	0.285
Cc1cccc(C=O)c1	3.223	2.483	0.465	0.140
Nc1ccc(C(=O)O)c(C(F)(F)F)c1	2.788	2.630	0.133	0.352
COc1ccc(C(=O)O)cc1C(F)(F)F	4.112	3.385	0.318	0.292
O=C1C=C(Cl)C(=O)c2ccccc21	3.226	2.486	0.113	0.190
Nc1ccc(Cl)cn1	3.943	3.540	0.335	0.386
Oc1ccc2ccccc2c1	0.953	2.537	0.047	0.845
N#Cc1cc(Cl)ccc1[N+](=O)[O-]	1.616	1.723	0.410	0.158
Nc1ccc(Br)cn1	5.588	5.376	0.316	0.396
CCCCOC(=O)C(=C)C	1.151	1.987	0.251	0.331
CCc1ccccc1C(=O)O	2.071	2.257	0.290	0.178
O=Cc1ccc(-c2ccncc2)cc1	4.855	5.356	0.207	0.638
Cc1cc(N)ccc1C(=O)O	3.566	3.480	0.289	0.377
Cc1ccc(Cl)cc1C(=O)O	0.582	0.960	0.087	0.135
Clc1cc(Br)ccn1	0.862	0.823	0.209	0.260
N#Cc1cc2ccccc2oc1=O	4.379	2.734	0.109	0.558
COc1ccc(B(O)O)c(OC)c1	4.370	3.835	0.172	0.464
Nn1c(-c2ccncc2)n[nH]c1=S	5.895	4.855	0.249	0.637
Nc1ccc(Cl)cn1	3.255	3.032	0.277	0.386
COc1c(C)cc(C(=O)O)cc1C	1.758	1.299	0.212	0.130
CCOC(=O)c1cc(OC)ccc1C	0.433	0.902	0.065	0.093
Clc1ccnc(Cl)c1	0.641	0.948	0.082	0.347
Brc1ccc2[nH]ccc2c1	1.500	0.765	0.086	0.108
O=C(Cl)Cc1ccccc1	0.494	0.393	0.019	0.072
C=Cc1ccc(OC(C)=O)cc1	2.101	2.116	0.195	0.584
Oc1cccc(Br)c1	0.789	0.867	0.046	0.178
O=Cc1ccccc1S(=O)(=O)O	3.086	1.937	0.096	0.463
COc1cc(N)c(C(=O)O)cc1OC	2.156	2.336	0.093	0.415
N#Cc1cccc2[nH]ccc12	1.623	1.673	0.076	0.269
N#Cc1cnc(Cl)cc1Cl	1.480	1.314	0.106	0.249
NC(N)=NS(=O)(=O)c1ccc(N)cc1	5.305	4.282	0.293	0.611
O=[N+]([O-])c1ccccc1Br	0.601	1.409	0.021	0.284
OCc1ccc(B(O)O)cc1	3.940	2.572	0.182	0.503
O=Cc1ccccc1F	0.623	0.686	0.088	0.186
CC(=O)c1ccccc1C(=O)O	1.658	1.444	0.236	0.219
CC(=O)Oc1cccc(C(=O)O)c1C	3.593	2.014	0.423	0.228
O=C(O)c1ccc(F)cc1C(F)(F)F	0.625	0.937	0.052	0.158
Nc1ccc(C(=O)O)c(Cl)c1	1.814	2.314	0.204	0.476
O=[N+]([O-])c1ccc(Cl)cc1	1.333	1.578	0.074	0.561
N#Cc1ccc(Br)cc1	2.622	2.372	0.101	0.303
O=CC=Cc1ccccc1	5.203	4.555	0.263	1.382
Cc1c(N)cccc1C(=O)O	1.007	1.502	0.063	0.268
O=Cc1ccc(-n2ccnc2)cc1	5.205	3.710	0.473	0.420
Nc1cccnc1Cl	1.046	0.996	0.132	0.145
CN(C)c1ccc(C(=O)O)cc1	3.037	5.758	0.253	0.934
N#Cc1ccc(O)cc1	3.821	3.200	0.698	0.343
COc1cccc(C(=O)O)c1	2.418	2.226	0.108	0.384
N#Cc1ccc(B(O)O)cc1	4.243	4.061	0.440	0.738
CC(=O)Nc1ccc(B(O)O)cc1	4.574	4.642	0.175	0.645
Nc1ccncc1N	1.157	1.229	0.084	0.211
COc1cc(Br)c(C(=O)O)cc1OC	0.849	1.647	0.069	0.198
Cc1c(Br)cccc1C(=O)O	1.728	1.880	0.111	0.253

COC(=O)C=CC(=O)OC	2.054	2.270	0.042	0.591
O=C1CCc2cc(Cl)ccc21	0.458	0.533	0.022	0.095
O=Cc1ccccc1Br	0.664	0.658	0.083	0.122
C1CCC(=CC1)C2=CC=CC=C2	0.210	0.725	0.018	0.205
COC(=O)c1ccc([N+](=O)[O-])cc1C	3.938	3.450	0.848	0.281
O=Cc1c(Cl)ncnc1Cl	0.820	1.524	0.034	0.330
COc1ccc(C(=O)O)c(Br)c1	0.902	1.422	0.051	0.250

Prediction error as a function of ensemble variance

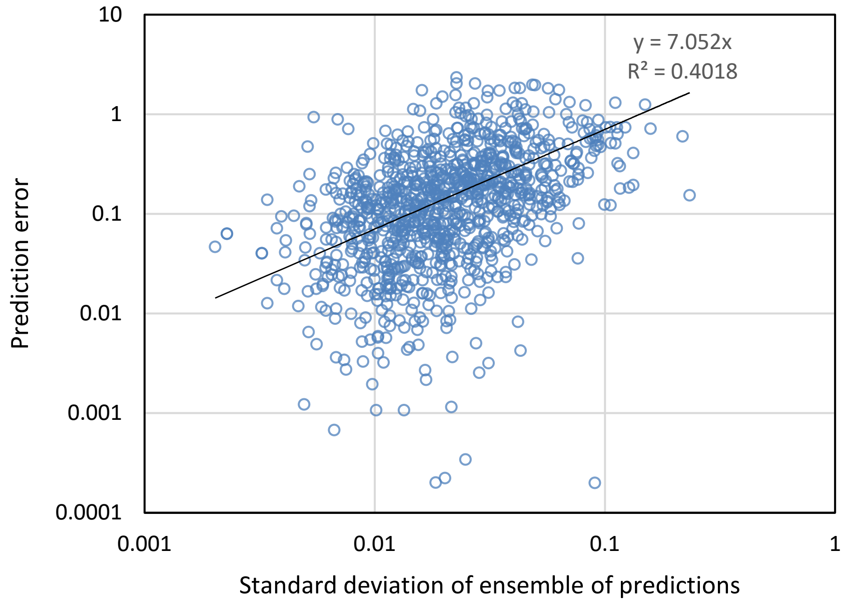

Figure S2. The error in the prediction of the $\log_{10}$ of the molar extinction coefficient versus the standard deviation of the prediction value, both normalized by the $\log_{10}$ of the molar extinction coefficient

The use of ensemble variance of a prediction as a means of determining the confidence in the value of that prediction is supported by the correlation between the variance (reported in figure S2 as standard error) and prediction error (reported as relative error). However, the correlation is not strong, making it difficult to *quantify* the confidence in the value.

Model scope and outliers

The outliers in this dataset are highlighted in figure S3. 4-(Dimethylamino)benzoic acid was severely overpredicted. Chemprop indicates that the overprediction is from the dimethylamino-benzene part of the molecule (purple), as this moiety is very common in dye molecules.

Indocyanine green also caused issues with the prediction of extinction coefficient, likely because it is very strongly absorbing and has a positive charge that can be delocalized over a large, conjugated substructure. Chemprop does not calculate partial charges on atoms, instead placing all the charge on the nitrogen atom indicated in the SMILES (and in figure S3), which leads to poor predictive power as the delocalization of charge is one of the keys to the molecule's optical properties. Luckily, structures like this are not very common in drug molecules (despite indocyanine green being used as a diagnostic stain for measuring blood flow).

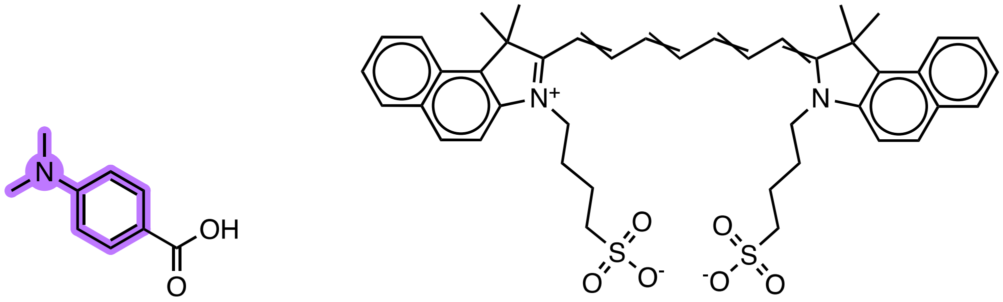

Figure S3. The structures of 4-(dimethylamino)benzoic acid (left) and indocyanine green (right). The part of the 4-(dimethylamino)benzoic acid molecule interpreted by chemprop as being important to its predicted extinction coefficient is highlighted in purple.

The prediction of insulin was also quite poor, as the training data excluded molecules with a molecular weight above 800 g/mol. Macromolecules such as proteins can have a huge range of molar extinction coefficients depending on the number of light-absorbing side chains present in the structure and folding of the macromolecular structure.

Analysis of test reactions

The peak associated with camostat, shown in Figure S4, is very broad compared to typical peaks. Figure S4 (top) shows the MS chromatogram at *m/z* = +399 (top) and the absorption chromatogram at 279 nm (bottom). The point in the top plot indicates the start of the MS peak with the correct mass to represent the target product. The closest lagging PDA peak is then integrated, the

integrated area is highlighted in orange. Compared to the other automatically extracted peaks (all marked with points), the camostat peak is very broad. The other peaks, from left to right, are guanidinobenzoic acid (low absorption at 279 nm), DMSO solvent, coupling agent byproduct, dimethylacetamide hydroxyphenylacetic acid ester, camostat, and internal standard.

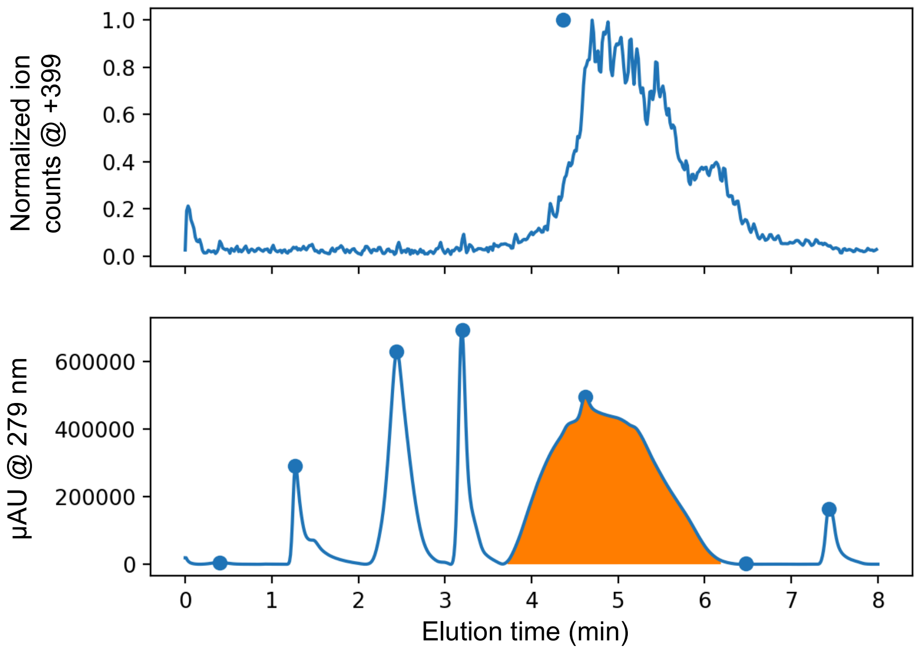

Figure S4. Chromatograms for the synthesis of camostat. MS chromatogram (top) at +399 m/z and PDA chromatogram (bottom) at 279 nm. Points indicated automatically extracted peaks. The orange area represents the area integrated of the camostat peak.

The synthesis of enalapril was found to result in a substantial amount of overreacted product under some reaction conditions (namely using EDC as coupling reagent as opposed to HATU). The over reacted product (shown in orange in Figure S5) has a nearly identical absorption spectrum as enalapril (in blue), causing a lower confidence in the resolution of their respective peaks by MCR. In the future, sensor fusion techniques[2] could enable use of MS data with the PDA data and curve resolution to better separate all compounds

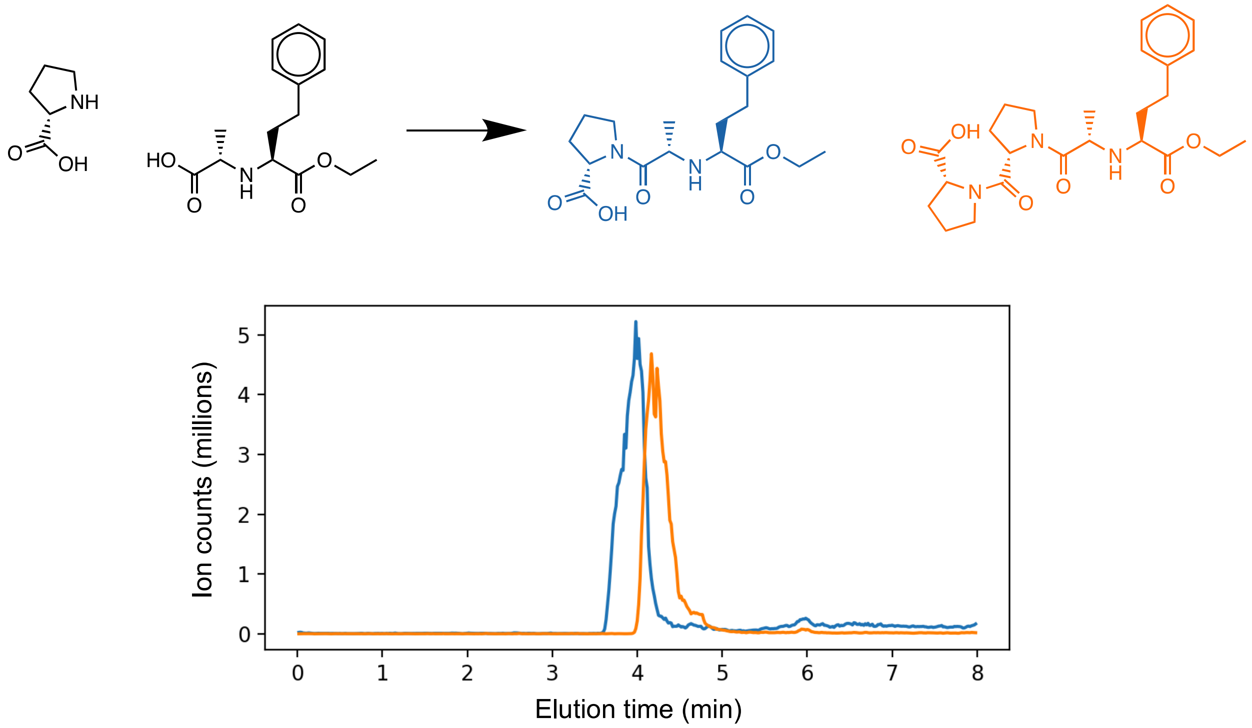

Figure S5. Reaction scheme (top) and MS chromatograms (bottom) from the synthesis of enalapril showing the target product (blue) and overreacted impurity (orange). The products overlap substantially, but not so much that they could not be resolved by MCR. The performance of MCR was not as robust as for other compounds because the product and impurity have nearly identical absorption spectra.

Reproducing figures 3 and 4 with provided data and HPLC_analysis.py

The discussed method was designed as an integrated part of the automated molecular discovery platform detailed in Koscher *et al.*[3] As part of the platform, the data generated by the HPLC were automatically analyzed in real time, that is, a reaction was analyzed as soon as the HPLC finished running that sample. To achieve this level of integration, the method as implemented within the platform makes use of a proprietary API provided by Shimadzu to control the HPLC instrument. Since access to this API is restricted, we have provided a version of the code that performs the same analysis, but on preexisting data. This code, contained in HPLC_analysis.py, can be used to recreate figures 3 and 4 with the chemprop model built using Reaxys data. We provided the data plotted in figures 3 and 4 in Figure3_data.xlsx and Figure4_data.xlsx, respectively. Using a

different chemprop model, such as the Deep4Chem model, will produce slightly different scatter plots on account of differences in predicted extinction coefficients.

References

1. Bessemans, L.; Jully, V.; de Raikem, C.; Albanese, M.; Moniotte, N.; Silversmet, P.; Lemoine, D., Automated Gravimetric Calibration to Optimize the Accuracy and Precision of TECAN Freedom EVO Liquid Handler. *SLAS Technology* **2016,** *21* (5), 693-705.
2. de Oliveira, R. R.; Avila, C.; Bourne, R.; Muller, F.; de Juan, A., Data fusion strategies to combine sensor and multivariate model outputs for multivariate statistical process control. *Analytical and Bioanalytical Chemistry* **2020,** *412* (9), 2151-2163.
3. Koscher, B. A.; Canty, R. B.; McDonald, M. A.; Greenman, K. P.; McGill, C. J.; Bilodeau, C. L.; Jin, W.; Wu, H.; Vermeire, F. H.; Jin, B.; Hart, T.; Kulesza, T.; Li, S.-C.; Jaakkola, T. S.; Barzilay, R.; Gómez-Bombarelli, R.; Green, W. H.; Jensen, K. F., Autonomous, multiproperty-driven molecular discovery: From predictions to measurements and back. *Science* **2023,** *382* (6677), eadi1407.
